# Epidemiology and risk stratification of low-grade gliomas in the United States, 2004-2019: A competing-risk regression model for survival analysis

**DOI:** 10.3389/fonc.2023.1079597

**Published:** 2023-03-01

**Authors:** Junguo Cao, Weijia Yan, Zhixin Zhan, Xinyu Hong, Hong Yan

**Affiliations:** ^1^ Shaanxi Eye Hospital (Xi’an People’s Hospital), Affiliated Xi'an Fourth Hospital, Northwestern Polytechnical University; Affiliated Guangren Hospital, School of Medicine, Xi’an Jiaotong University, Xi’an, Shaanxi, China; ^2^ Division of Experimental Neurosurgery, Department of Neurosurgery, University of Heidelberg, Heidelberg, Germany; ^3^ Department of Ophthalmology, University of Heidelberg, Heidelberg, Germany; ^4^ Department of Neurosurgery, The First Hospital of Jilin University, Changchun, China

**Keywords:** SEER Program, low-grade glioma, incidence, risk factor, molecular marker

## Abstract

**Background:**

Understanding the epidemiology and prognostic factors of low-grade gliomas (LGGs) can help estimate the public health impact and optimize risk stratification and treatment strategies.

**Methods:**

3 337 patients diagnosed with LGGs were collected from the Surveillance, Epidemiology, and End Results (SEER) dataset, 2004–2019. The incidence trends of LGGs were analyzed by patient demographics (sex, age, race, and ethnicity). In addition, a competing risk regression model was used to explore the prognostic factors of LGGs by patient demographics, tumor characteristics (histological subtypes, invasiveness, and size), treatment modality, and molecular markers (IDH mutation and 1p/19q codeletion).

**Results:**

LGGs occurred more frequently in male, non-Hispanic, and White populations. The incidence rate of mixed gliomas was stable from 2004 to 2013 and decreased dramatically to nearly zero until 2019. The risk of death increased 1.99 times for every 20-year increase in patient age, and 60 years is a predictive cut-off age for risk stratification of LGGs. Male patients showed poorer LGG-specific survival. Among the different subtypes, astrocytoma has the worst prognosis, followed by mixed glioma and oligodendroglioma. Tumors with larger size (≥5 cm) and invasive behavior tended to have poorer survival. Patients who underwent gross total resection had better survival rates than those who underwent subtotal resection. Among the different treatment modalities, surgery alone had the best survival, followed by surgery + radiotherapy + chemotherapy, but chemotherapy alone had a higher death risk than no treatment. Furthermore, age, invasiveness, and molecular markers were the most robust prognostic factors.

**Conclusion:**

This study reviewed the incidence trends and identified several prognostic factors that help clinicians identify high-risk patients and determine the need for postoperative treatment according to guidelines.

## 1 Introduction

Low-grade gliomas (LGGs) constitute a heterogeneous group of neuroepithelial tumors that arise from astrocytes of the central nervous system (CNS), accounting for approximately 30% of childhood and 6.4% of adult primary CNS tumors (1–4). Classically, LGGs contain World Health Organization (WHO) grade 1 and 2 gliomas (5). They are benign and slow-growing, but many are rarely cured and frequently transform into higher-grade tumors (6). Although LGGs encompass many distinct subtypes, the most commonly used in the literature include astrocytoma, oligodendrogliomas, and mixed gliomas, which comprise the majority of LGGs (5). Understanding the epidemiology of LGG can help estimate its public health impact; however, population-based surveys on incidence trends of LGGs are uncertain and controversial because trends change as the diagnosis evolves and histological criteria and classification schemes are modified (7). This study updated LGG incidence trends based on a comprehensive analysis of the most recent national dataset, which is more reliable, representative, and up-to-date.

The clinical effects of LGGs are variable, with survival ranging from less than 2 years to more than 10 years (8). Patient demographics, tumor characteristics, treatment modalities, and molecular alterations contribute to this variability (8, 9). Due to diffuse infiltration, complete surgical resection of LGGs is not always feasible (10). Optimal treatment strategies have long been controversial because of difficulties in identifying high-risk LGG patients (11, 12). Researchers have identified many prognostic factors for LGGs, such as age, histological subtype, tumor diameter, and extent of resection, which are critical for risk stratification and individualized therapy (3, 13). However, current research has only included small cases or analyzed overall survival (OS), which has many statistical drawbacks and does not consider competing risks, tends to produce inaccurate estimates (14–16). Patients with LGG generally have a good prognosis and long survival, which increases the risk of death from other causes, also known as competing risks, such as heart attacks or traffic accidents. Furthermore, previous studies have revealed that OS is correlated with age and sex. However, the patient’s demise might also be caused by an illness specific to men or an increase in age-related disorders as they affect older patients (17). Therefore, it is necessary to expand the sample size and use a competing risk model to minimize the drawbacks of overall survival.

The Surveillance, Epidemiology, and End Results (SEER) program is a reliable source of data on cancer incidence and survival in the US. Approximately 48.0% of the U.S. population is presently covered by the SEER dataset. The International Classification of Diseases for Oncology, Third Edition (ICD-O-3) was used to code tumor cases. In the current investigation, the SEER registry was applied to conduct a thorough analysis of LGG patients in the United States from 2004 to 2019 (18). The occurrence of LGGs in relation to patient demographics, including age, sex, race, and ethnicity, was thoroughly reviewed in this study. We also examined prognostic indicators based on patient demographics (age, sex, ethnicity, and race), tumor characteristics (histological subtypes, size, and invasiveness), therapeutic approach (extent of resection and treatment modality), and molecular markers (IDH mutations and 1p/19q codeletion). Here, we used a competing risk regression model to examine the specific survival of LGGs and comprehend the contributions of these variables to the death of LGGs.

## 2 Materials and methods

### 2.1 Data collection

The SEER database “SEER 17 Regs Plus Nov 2021 Sub (2000-2019)” was used to collect LGGs cases from 2004 to 2019 without an age restriction. Following ICD-O-3 codes allowed for the identification of LGGs: 9382/3, 9384/1, 9400/3, 9410/3, 9411/3, 9412/1, 9413/0, 9420/3, 9421/1, 9424/3, 9425/3, 9431/1, and 9450/3. Subjects without primary or initial tumors and those with undefined or missing data were excluded. A cohort of 13 337 patients was obtained using all screening criteria.

### 2.2 Incidence trend analysis

For LGGs from 2004 to 2019, age-adjusted incidence rates (IRs) and 95% confidence intervals (CIs) were calculated based on age, sex, race, and ethnicity. All items were split according to the 5-year intervals of the individuals’ ages. White, Black, Asian/Pacific Islander (API), and American Indian/Alaska Native (AIAN) were the four race classifications. The categories for ethnicity contained non-Hispanic and His-panic. Comparisons of IR excluded categories that were not specified or unknown. In this study, age-adjusted IRs were reported per 100,000 people and normalized to the US population in 2000. The SEERStat program was used to compute the IRs. The Join-point Regression Program was employed to determine the annual percentage change (APC). For the APCs permutation test, statistical significance was defined as *P* < 0.01. All graphs were produced using GraphPad Prism 7.0.

### 2.3 Competing risk analysis

>Survival studies by sex, age, race, ethnicity, histological subtypes, invasiveness, tumor size, extent of resection, and treatment modalities were conducted. Five age ranges (0-19, 20-39, 40-59, 60-79, and 80+ years) and two tumor size ranges (<5 cm and ≥5 cm) were used. Histological types include astrocytoma, oligodendrogliomas, and mixed gliomas. To make it clear, “LGGs” refers to all subtypes of LGG; otherwise, specific subtype designations are used in this paper. Invasiveness was categorized as non-invasive and invasive. Surgery type was divided into three groups: no surgery (NS), gross total resection (GTR) and subtotal resection (STR). In addition, no treatment (No), surgery only, chemotherapy (CT) only, surgery + radiation (RT), surgery + CT, and surgery + RT + CT were the six categories comprising the treatment modality. Due to the limited sample size, LGGs in the AIAN population were excluded from survival analyses.

Competitive risk analysis is a unique form of survival analysis that attempts to accurately assesses the marginal probability of an event in the presence of competitive events. Traditional methods for describing the survival process, such as the Kaplan-Meier model, do not consider multiple competing causes of the same event. As a result, these methods often produce inaccurate estimates when analyzing the marginal probabilities of cause-specific events. The cumulative incidence function (CIF), which estimates the marginal probability of a single event as a function of its cause-specific and overall survival probabilities, is a working method for addressing this problem. In this study, the cumulative incidence of LGG-related mortality was calculated for every variable after considering mortality from other causes. The Fine-Gray model was used for competing risk analysis to compute the sub-distribution hazard ratio (SHR) and 95% CIs. Internal validation was performed by bootstrap analysis based on 1000 bootstrap times. In addition, *P* < 0.01 was used to define statistical significance. The SAS program and IBM SPSS Statistics 25 was used for data processing.

## 3 Results

### 3.1 Baseline patient characteristics

Data from a total of 13 337 LGG patients were investigated. Patient demographics, tumor characteristics, treatment modalities, and molecular markers of the analyzed cases are presented in **Table 1** and **Supplementary Table 1**. The age group with the most patients was 40-59 years, including 4270 (32.0%), followed by 20-39 years with 4181 (31.4%), 80+ years with 2432 (18.2%), and 00-19 years with 2047 (15.4%) patients. Male patients comprised 56.3% (7513) of the total population, while female patients accounted for 43.7% (5824). In addition, White patients comprised the majority (11320, 84.9%), followed by Black patients (893, 6.7%) and API patients (862, 6.5%). Overall, 83.5% (11142) were non-Hispanic. For the glioma histological subtypes, the largest group was astrocytoma, with 5245 (39.3%) patients, followed by oligodendroglioma (3298, 24.7%) and mixed glioma (1890, 14.2%). Regarding invasiveness, most cases were non-invasive (9913, 74.3%). Most tumors were < 5 cm in size (5966, 44.7%), and tumors ≥ 5 cm accounted for 24.7% (3291). Among the surgery types, 23.8% of patients underwent STR, and 30.8% of patients underwent GTR. Regarding treatment modality, 36.2% of the patients underwent surgery only, 9.0% only received chemotherapy, 9.3% underwent surgery and radiotherapy, 5.3% under-went surgery and chemotherapy, and 21.3% underwent surgery, radiotherapy, and chemotherapy.

**Table 1:** Patient baseline characteristics.

Characteristics	Subgroups	Number (%)	Rate
Age	00-19 yrs	2047	15.35
	20-39 yrs	4181	31.35
	40-59 yrs	4270	32.02
	60-79 yrs	2432	18.23
	80+ yrs	407	3.05
Sex	Female	5824	43.67
	Male	7513	56.33
Race	White	11320	84.88
	Black	893	6.70
	AIAN	133	1.00
	API	862	6.46
Ethnicity	Hispanic	2195	16.46
	non-Hispanic	11142	83.54
Subtype	Astrocytoma	5245	39.33
	Oligodendroglioma	3298	24.73
	Mixed glioma	1890	14.17
	Others	2904	21.77
Invasiveness	Non-invasive	9913	74.33
	Invasive	319	2.39
Size	< 5 cm	5966	44.73
	≥ 5 cm	3291	24.68
Surgery type	NS	5830	43.71
	STR	3169	23.76
	GTR	4102	30.76
Treatment modality	No	2406	18.04
	Surgery	4823	36.16
	CT	1202	9.01
	Surgery + RT	1234	9.25
	Surgery + CT	706	5.29
	Surgery + RT + CT	2841	21.30

AIAN, American Indian/Alaska Native; API, Asian/Pacific Islander; CT, chemotherapy; GTR, gross total resection; NS, no surgery; No, no treatment; STR, subtotal resection; RT, radiotherapy.

### 3.2 Incidence trends of LGGs by age, sex, race, ethnicity, and histological subtype

**Figure 1:**
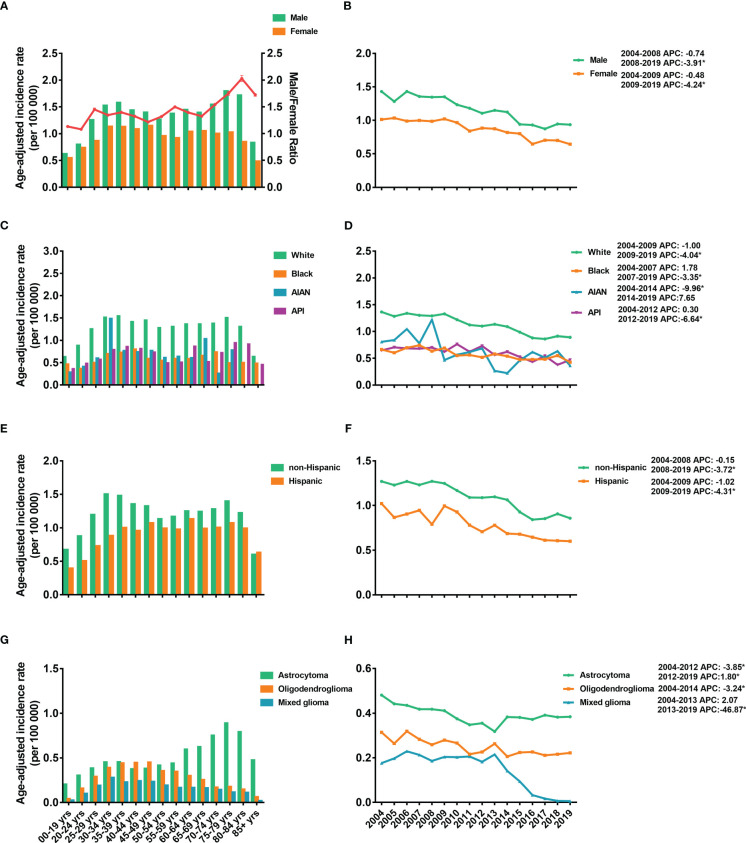
Age-adjusted incidence rates (IRs) and annual percent changes (APCs). IRs by 5-year age intervals and by sex **(A)**, race **(C)**, ethnicity **(E)**, and histological subtypes **(G)**. APCs by sex **(B)**, race **(D)**, ethnicity **(F)** and histological subtypes **(H)** over time from 2004–2019. * *P* < 0.01.

The age-adjusted IRs for the LGGs from 2004 to 2019 are shown in **Figure 1**. From the data, LGGs had an average IR of 1.12 (95% CI: 1.03–1.21) per 100,000 population. The IR of females was lower than that of males, and the male-to-female ratio of the IR increased with age, peaking at 2.03 (95% CI: 1.97–2.09) in the 80–84-year-old group (**Figure 1A**). The IRs were stable from 2004-2008 for males and 2004-2009 for females, followed by a significant decrease until 2019 (**Figure 1B**). The White population has the highest IRs compare to other races, 1.25 per 100,000 [95% CI: 1.15–1.36], followed by 0.67 [95% CI: 0.48–0.92] in API population, 0.60 [95% CI: 0.26–1.62] in AIAN population, and 0.58 [95% CI: 0.40–0.82] in Black population (**Figure 1C**). IRs showed a decreasing trend in all races (**Figure 1D**). For IR by ethnicity, the overall IR of the non-Hispanic population (1.05 [95% CI: 0.96–1.15]) was higher than that of Hispanics (0.76 [95% CI: 0.60–0.97]) (**Figures 1E, F**).

Regarding the incidence of different subtypes, astrocytoma demonstrated the highest IR, 0.43 (95% CI: 0.38-0.49) per 100,000 population, followed by 0.21 (95% CI: 0.18-0.25) for oligodendrogliomas and 0.12 (95% CI: 0.10-0.15) for mixed gliomas (**Figure 1G**). Furthermore, the IRs of astrocytoma increased with age and reached a peak IR of 0.89 (95% CI: 0.78-1.00) at age 75-79 years. However, the IRs of oligodendrogliomas and mixed gliomas peaked at younger ages, 45-49 years (0.45 [95% CI: 0.41-0.50]) and 40-44 years (0.24 [95% CI: 0.21-0.28]), respectively (**Figure 1G**). The IRs of astrocytoma dropped from 2004-2012 and then increased significantly from 2012-2019 (**Figure 1H**). For oligodendroglioma, IR decreased from 2004-2014 and kept stable between 2014-2018. However, the IRs of the mixed glioma dropped obviously from 2013-2019 (**Figure 1H**).

### 3.3 Cause-specific survival of patients with LGGs

#### 3.3.1 Cumulative incidence analysis

**Figure 2:**
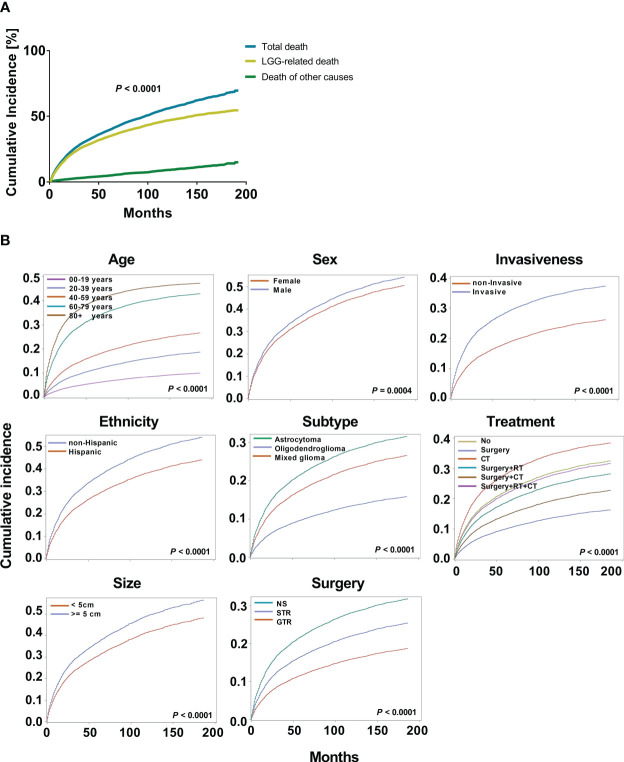
Cumulative incidence curves for low grade gliomas (LGGs). **(A)**. The cumulative incidence total death and death from LGGs and those from other causes. **(B)**. The cumulative incidence of tumor-related death was computed for each factor after accounting for the death of other causes. The subsequent plots present fraction of the population that has died from their LGGs by age, sex, ethnicity, histological subtypes, invasiveness, tumor size, surgery, and treatment modality.

Kaplan-Meier and multivariate Cox proportional hazard models were used to explore the risk factors of overall survival (**Supplementary Figure 1**). To further optimize survival analysis and avoid the limitations of overall survival, a competing risk regression model was used to perform LGG-specific survival analysis. At data collection, 4759 patients with LGG (35.7%) died of their gliomas. When we compared the cumulative incidence of deaths from LGGs with all-cause mortality, the data demonstrated a significant difference (*P* < 0.0001, **Figure 2A**). Additionally, the cumulative risk of mortality from LGG was 14.6% (95% CI: 10.6%-14.3%), 34.7% (95% CI: 29.2%-35.0%), and 46.8% (95% CI: 35.2%-42.0%) at 1, 5, and 10 years, respectively. Age (*P* < 0.0001), sex (*P* = 0.0004), ethnicity (*P* < 0.0001), tumor subtype (*P* < 0.0001), invasiveness (*P* < 0.0001), tumor size (*P* < 0.0001), surgery type (*P* < 0.0001) and treatment modality (*P* < 0.0001) showed significant variations in cause-specific survival for LGGs (**Figure 2B**).

#### 3.3.2 Factors associated with cause-specific survival based on a competing risk regression model

**Figure 3:**
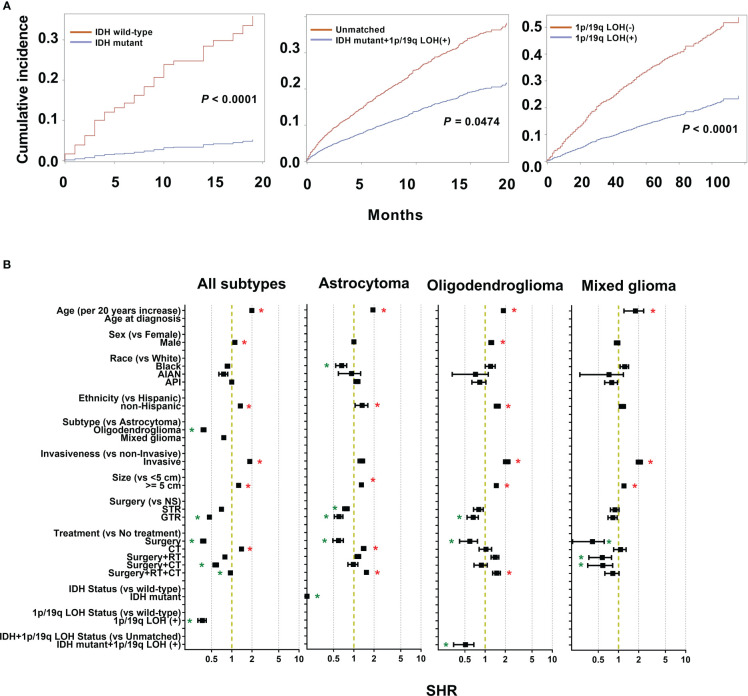
Cumulative incidence curves and sub-distribution hazard ratios (SHR) for each of the features of the survival model. **(A)** The subsequent plots present fraction of the population that has died from low grade gliomas (LGGs) by IDH mutations and 1p/19q codeletion status. **(B)** SHR by patient demographics (sex, age, race, and ethnicity), tumor (histological subtypes, invasiveness, size), treatment (extent of resection and treatment modality), and molecular markers (IDH mutation and 1p/19q codeletion status) in competing risk regression model for LGG-specific survival. * *P* < 0.01.

After competing risk analysis, the SHRs for the risk of dying from LGG are displayed in **Figure 3B** and **Supplementary Table 2**. Age, sex, ethnicity, historical subtype, invasiveness, tumor size, extent of resection, and therapy modality all significantly influenced CSS. For every additional 20 years of patient age, the average risk of mortality rises by 1.99 times (SHR: 1.99 [95% CI: 1.97-2.00], *P* < 0.001). Males had an 11.1% higher mortality risk than females (SHR: 1.11 [95% CI: 1.10-1.13], *P* = 0.0004). Patients who were non-Hispanics had a 34.2% higher risk of mortality, but not supported by bootstrap analysis. Patients with oligodendroglioma exhibited a 61.9% decreased mortality risk compared to those with astrocytoma (SHR: 0.38 [95% CI: 0.35-0.41], *P* < 0.001). Compared to non-invasive tumors, invasive tumors have a mortality risk that is 85.3% greater (SHR: 1.85 [95% CI: 1.82-1.89], *P* < 0.001). In comparison to individuals with small tumors (< 5 cm), those with large tumors (≥ 5 cm) had a 26.2% greater risk of mortality (SHR: 1.26 [95% CI: 1.24-1.28], *P* < 0.0001). GTR decreased the risk of mortality by 53.6%. Additionally, the mortality risk decreased by 62.3% and 4.6% in patients who underwent surgery alone and surgery + RT + CT, respectively (surgery: SHR: 0.38 [95% CI: 0.35–0.41], *P* < 0.001; surgery + RT+ CT: SHR: 0.95 [95% CI: 0.93–0.98], *P* < 0.001). However, compared to patients who received no therapy, those who underwent CT had a 39.5% higher mortality rate (HR: 1.40 [95% CI: 1.36–1.43], *P* < 0.001).

### 3.4 Cause-specific survival of patients with astrocytoma, oligodendroglioma, and mixed glioma

#### 3.4.1 Cumulative incidence analysis

**Figure 4:**
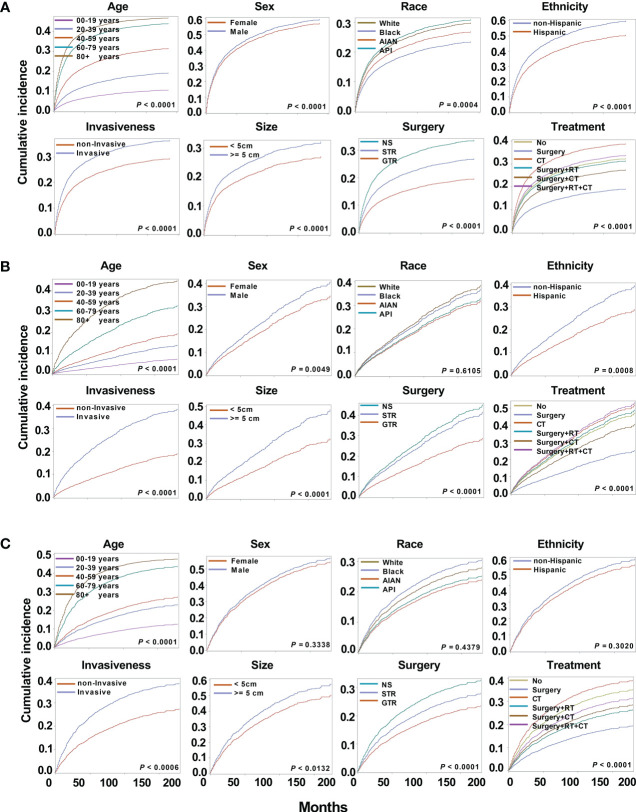
Cumulative incidence curves for astrocytoma, oligodendrogliomas, and mixed gliomas. The subsequent plots present fraction of the population that has died from their astrocytoma **(A)**, oligodendrogliomas **(B)**, and mixed gliomas **(C)** by age, sex, race, ethnicity, invasiveness, tumor size, surgery, and treatment modality.

Among the different subtypes, oligodendroglioma has the lowest incidence risk, followed by mixed glioma, whereas astrocytoma has the highest risk. Therefore, there is a huge difference in the survival of different subtypes, and it is necessary to perform survival analysis by subtype. As a result of astrocytoma, substantial variations in cumulative risk were demonstrated by all eight variables: age (*P* < 0.0001), sex (*P* < 0.0001), race (*P* = 0.0004), ethnicity (*P* < 0.0001), invasiveness (*P* < 0.0001), tumor size (*P* < 0.0001), surgery type (*P* < 0.0001) and treatment modality (*P* < 0.0001) (**Figure 4A**). Furthermore, except for race (*P* = 0.6105) in oligodendrogliomas, sex (*P* = 0.3338), race (*P* = 0.4379), and ethnicity (*P* = 0.3020) in mixed gliomas, all other variables may be potential prognostic factors (**Figures 4B, C**).

#### 3.4.2 Factors associated with Cause-specific survival based on a competing risk regression model

From the results of the competing risk regression using Fine-Gray model and internal validation by bootstrap analysis, age, race, ethnicity, tumor size, surgery type, and treatment modality had a substantial impact on CSS for astrocytoma; age, invasiveness, size, surgery type, and treatment modality for oligodendrogliomas; age and treatment modality for mixed gliomas (**Figure 3B** and **Supplementary Table 2**). Specifically, compared with low-grade astrocytoma, oligodendroglioma patients had a 61.9% decreased mortality risk (SHR: 0.38 [95% CI: 0.35–0.41], *P* < 0.001). Mixed gliomas also shown a 24.0% decreased mortality risk, although the difference was not significant. In detail, the risk of mortality rises 1.92 times for every additional 20 years of age for astrocytoma patients (SHR: 1.92 [95% CI: 1.90–1.94], *P* < 0.001), 1.87 times for oligodendroglioma (SHR: 1.87 [95% CI: 1.82–1.92], *P* < 0.001), and 1.78 times for mixed glioma patients (SHR: 1.78 [95% CI: 1.21–2.36], *P* < 0.001).

For astrocytoma, Black patients showed 34.2% lower risk of death compared to White (SHR: 0.66 [95% CI: 0.54–0.78], *P* = 0.0006). Furthermore, the mean age was 45.49 ± 1.56 years for White patients and 38.71± 0.10 years for Black ones, which was statistically different (*P* < 0.001). In further survival analysis, an interaction analysis was performed by including the age and race, and the results showed no statistical difference in survival of the two groups (*P* = 0.5477). Therefore, the data supported that the younger age of Black patients may contribute to longer survival. For the ethnicity of patients, non-Hispanic patients have a 32.9% (SHR: 1.33 [95% CI: 1.05–1.61], *P* = 0.001) greater risk of death for astrocytoma. The death risk of invasive oligodendroglioma was 2.11 (SHR: 2.11 [95% CI: 1.88–2.33], *P* = 0.0009) times that of non-invasive tumors. Large oligodendroglioma patients (≥ 5 cm) had a 46.8% greater risk of mortality (SHR: 1.47 [95% CI: 1.37–1.57], *P* < 0.001) than those with small tumors (< 5 cm). This was followed by 29.5% (SHR: 1.30 [95% CI: 1.23–1.36], *P* < 0.001) in astrocytoma.

Regarding different surgery types, GTR reduced the death risk by 40.1% (SHR: 0.60 [95% CI: 0.51–0.69], *P* < 0.001) for astrocytoma, and 33.6% (SHR: 0.66 [95% CI: 0.54–0.79], *P* = 0.0013) for oligodendrogliomas. For astrocytoma, patients who under-went STR had a 23.3% (SHR: 0.77 [95% CI: 0.69–0.85], *P* = 0.0012) lower risk of death. About treatment modalities, surgery alone reduced the death risk by 40.8% (SHR: 0.59 [95% CI: 0.49–0.70], *P* < 0.001), 40.8% (SHR: 0.59 [95% CI: 0.42–0.77], *P* = 0.0028), and 59.1% (SHR: 0.41 [95% CI: 0.21–0.61], *P* < 0.001) in astrocytoma, oligodendroglioma, and mixed glioma, respectively. In addition, surgery + RT decreased the risk of death by 42.7% (SHR: 0.57 [95% CI: 0.36–0.79], *P* = 0.0086) in mixed glioma. However, astrocytoma patients who underwent CT and surgery + RT +CT had a 40.1% (SHR: 1.40 [95% CI: 1.30–1.50], *P* = 0.0006) and 54.0% (SHR: 1.54 [95% CI: 1.45–1.64], *P* < 0.001) increased risk of death compared to no treatment. In oligodendrogliomas, the surgery + RT +CT group also had a higher risk of death (SHR: 1.49 [95% CI: 1.29–1.68], *P* = 0.0416), but without statistical significance.

### 3.5 Significance of IDH mutations and 1p/19q codeletion in the survival of LGG patients

From 5245 astrocytoma patients, IDH mutation status was recorded in 489 cases, including 199 (40.7%) IDH wild-type cases and 290 (59.3%) IDH-mutant cases. Compared to wild-type astrocytoma, IDH-mutant patients had longer survival (**Figure 3A**). Furthermore, competing risk analysis showed that IDH-mutant patients had an 87.9% (SHR: 0.12 [95% CI: 0.01–0.31], *P* < 0.001) lower risk of death (**Figure 3B**). For oligodendroglioma, 330 cases had both IDH mutations and 1q/19q codeletion; others are defined as unmatched cases, including unknown, wild-type, IDH mutation, or 1p/19 codeletion. The IDH-mutation + 1q/19q codeletion patients had a decreased cumulative incidence of death compared to the unmatched patients (**Figure 3A**). In addition, the IDH mutation+ 1q/19q codeletion group had a 49.1% (SHR: 0.51 [95% CI: 0.34–0.68], *P* = 0.0474) lower risk of death (**Figure 3B**), but without significant differences. Of all collected LGG cases, 2111 recorded the 1q/19q LOH status, 1050 (49.7%) presented 1q/19q codeletion, and 1061 (50.3%) did not. Based on the results of CSS analysis, patients with 1q/19q codeletion had longer survival (**Figure 3A**). Furthermore, LGGs patients with 1q/19q codeletion had a 63.6% (SHR: 0.36 [95% CI: 0.31–0.42], *P* < 0.001) lower risk of death than others (**Figure 3B**).

To exclude the potential effects of the treatment regimens in this study, the interaction effect of the treatment regimens and molecular markers was considered. However, we only observed that LGGs with 1q/19q codeletion showed significantly longer survival by interaction analysis (*P* = 0.0069), but not for IDH-mutant in astrocytoma patients (*P* = 0.9664), and IDH-mutation + 1q/19q codeletion oligodendroglioma patients (*P* = 0.0616). Taken together, after considering the possible interaction effects of treatment regimens, IDH mutation in LGGs could be a critical prognostic factor.

### 3.6 Patient age, tumor aggressiveness, and molecular markers may be robust prognostic factors in risk stratification of LGG patients

**Figure 5:**
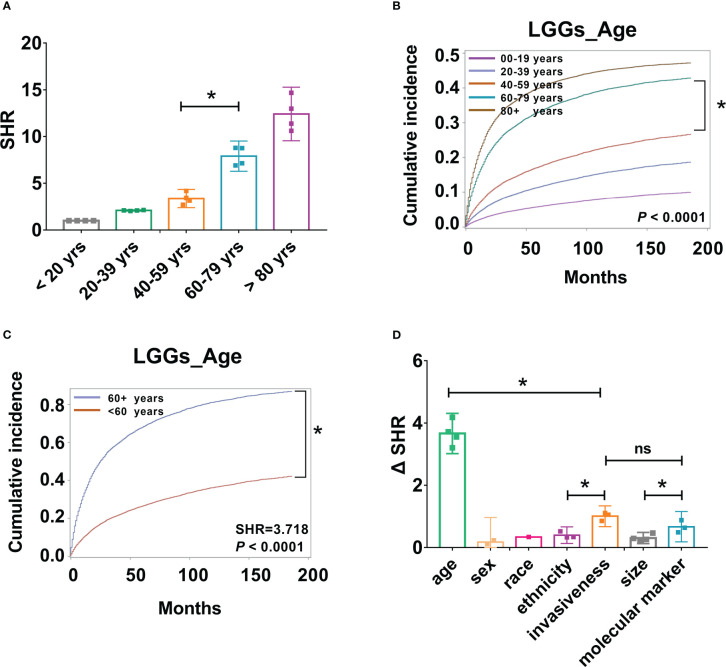
Patient age, tumor aggressiveness, and molecular markers may be important factors in risk stratification of LGG patients. **(A)**. SHR from four groups, including all LGGs, astrocytoma, oligodendrogliomas, and mixed gliomas across different age groups were compared, *P < 0.001. **(B)**. Cumulative incidence curves of LGGs by five age groups, 0-19, 20-39, 40-59, 60-79, and 80+ years, *P < 0.001. **(C)**. Cumulative incidence curves of LGGs by two age groups, < 60, and 60 + years, * *P* < 0.001. **(D)**. Absolute differences in SHR (ΔSHR) between each reference and subtype were calculated and compared across different factors, including age, sex, race, ethnicity, invasiveness, size, and molecular markers. ns, no significant differences.

The above results demonstrated that age is a critical risk factor for LGG-specific survival, and the risk of mortality increases by approximately two-fold for every additional 20 years of age for all types of LGGs. To further determine the most predictive cut-off points for LGG-specific mortality risk stratification, SHR across different age groups were compared. SHRs of the four groups, including all LGGs, astrocytoma, oligodendrogliomas, and mixed gliomas, were included. We observed a substantial in-crease in SHR from 40-59 years to 60-79 years group (*P* < 0.001, **Figure 5A**); however, the increases in SHR from 20-39 years to 40-59 years group, and from 60-79 years to 80 + years group were not significantly different (*P* > 0.001, **Figure 5A**). Furthermore, differences in the cumulative incidence of each age group were compared. Similarly, there was a significant increase in the cumulative incidence between the 40-59 years and 60-79 years group (*P* < 0.001, **Figure 5B**), which indicates that 60 years may be a predictive cut-off point for CSS. To validate this result, patient age was divided into two groups, < 60 years and 60 + years, and LGG-specific survival analysis was per-formed again. As a result, substantial variations in cumulative risk were observed be-tween patients aged < 60 and ≥ 60 years (*P* < 0.001, **Figure 5C**). The risk of mortality for older patients (≥ 60 years) are 3.72 (SHR: 3.72 [95% CI: 3.69–3.75], *P* < 0.0001) times that of younger patients (< 60 years).

Although several risk factors have been identified, the predictive effects of each factor are different. To improve the clinical significance, the absolute differences in SHR (ΔSHR) between each reference and subtype were calculated and compared across different factors. Patient age had the highest ΔSHR, with a value of 3.663 (ΔSHR:3.663 [95% CI:3.460-3.897]). This was followed by tumor invasiveness (ΔSHR: 1.004 [95% CI:0.927-1.081]) and molecular markers (ΔSHR: 0.6687 [95% CI:0.556-0.782]), which were significantly higher than the other variables (**Figure 5D**).

## 4 Discussion

LGG is a common CNS tumor; some are rarely cured and frequently transform into higher-grade tumors, presenting a substantial challenge to neurosurgeons. Understanding the epidemiology of LGGs can help estimate their public health impact, and identifying reliable prognostic factors can help optimize risk stratification. Herein, this study updated the incidence and survival trends of LGGs from 2004 to 2019 by using an extensive database. We observed that LGGs occurred more frequently in male, non-Hispanic, and White populations. Interestingly, the IR of mixed gliomas was stable from 2004 to 2013, but decreased dramatically to nearly zero until 2019, which is like a shunt to astrocytoma or oligodendrogliomas. The risk of death increased 1.99 times for every 20-year increase in patient age. Importantly, we identified that 60 years is a predictive cutoff age for risk stratification of LGGs. In addition, male, White, and non-Hispanic populations showed poorer LGG-specific survival rates. Astrocytoma has the worst prognosis among the different subtypes, followed by mixed gliomas and oligodendrogliomas. Tumors with larger size (≥5 cm) and invasive behavior tended to have poorer survival rates. Patients who underwent gross total resection had better survival rates than those who underwent subtotal resections. Among the different treatment modalities, surgery alone had the best survival, followed by surgery + chemotherapy and surgery + radiotherapy + chemotherapy; however, chemotherapy alone had a higher death risk than no treatment. Specifically, after comparing the SHRs of the different variables, age, invasiveness, and molecular markers were regarded as the most robust prognostic factors. Therefore, this study updated the incidence trends of LGGs and identified the most powerful prognostic factors for risk stratification in patients with LGGs.

### 4.1 Incidence trends

In this research, we gathered information on 13 337 cases of LGGs and identified that the patients are mainly between the ages of 40-59 years (32.0%) and 20-39 years (31.4%). Furthermore, the IR was stable at 1.07-1.36 cases per 100 000 populations in different age groups from 25-84 years. Unlike meningioma, in which IRs climbed with age, and most patients were over 60 years, the correlation between the incidence of LGGs and age is not obvious. Although the IR was 0.68 cases per 100 000 populations in patients of 0-24 years, which is lower than in adult patients, LGG is the most common children brain tumor and accounts for 30% of all CNS tumors in children (4). Regarding sex, most of the patients are male (56.3%), and the IR of males was always higher than females. Additionally, we discovered that the ratio of LGG IRs for men to women was 1.43:1 and that it elevated with age, peaking at 2.03 in the 80-84-year-old group. It is possible that uncertain sex-related variables have a significant influence. Further research is necessary to elucidate the potential mechanisms. Most patients were White (84.9%) and non-Hispanic (83.5%) and the reason for this racial or ethnic discrepancy remains uncertain. This may be due to testing bias, genetic variation, or some uncertainty (12).

Regarding incidence trends of LGGs by sex, age, race, and ethnicity, the IRs were stable from 2004 to 2009 and then dropped until 2019. Changes in the incidence of LGGs may contribute to the modifications of classification guideline published in 2007 (2). However, the IRs of mixed gliomas dropped obviously from 2013-2019, but the IR of astrocytoma increased significantly from 2012-2019, and the IR of oligodendrogliomas decreased from 2004-2014 but keep stable until 2019. Regarding the altered distribution of LGGs, Sahm et al. discovered that when combining the molecular and cytogenetic criteria, mixed gliomas with intermediated astrocytic and oligodendroglial appearance can be recategorized as either astrocytoma or oligodendrogliomas, which may explain these trends (7, 19). Furthermore, the IR of mixed gliomas decreased dramatically to nearly zero, which reminds neuropathologists to use molecular markers for diagnosis when encountering mixed gliomas because it is likely to confuse the other two tumors, astrocytoma and oligodendrogliomas.

### 4.2 Survival analysis

From the survival analysis, we discovered several patient demographics linked with worse prognoses in LGG patients. For the CSS of LGG patients, older age, especially beyond 60 years, was a significant contributor to a poorer outcome. This is in line with the findings of multiple studies showing that old age is a risk factor for poor survival (20, 21). It is currently debatable whether age should be studied as a continuous or binomial variable. Previous research has downplayed the detrimental effect of growing older on prognosis, particularly after the age of 60 years (22). According to the study, the death rate increased by 23% for each year when a patient was older than 60 years at the time of diagnosis (22). Pignatti et al. discovered a similar linear relationship between the prognosis and age (13). In line with the present study, we observed that for every 20-year increase in patient age, the probability of mortality increased by 1.99 times. However, an absolute cutoff point is convenient for clinicians to distinguish between older and young patients. Various cut-off ages have been applied in different studies without evidence. Pignatti et al. used an age threshold of 40 years, Kumthekar et al. regarded over 50 years as older LGG patients, and Capelle et al. found that independent factors for poor prognosis in LGG included age ≥ 55 years (13, 23, 24). Herein, we compared SHR across different age groups and identified that 60 years could be a predictive cut-off age. This was further confirmed by CSS analysis, and the risk of morality for older patients (60 + years) is 3.72 (SHR: 3.72 [95% CI: 3.69–3.75], *P* < 0.0001) times that of young patients (< 60 years).

The male population with LGGs showed poorer CSS than females. The incidence and survival of brain tumors, such as glioblastomas, fluctuate depending on the gender, with men experiencing a greater incidence rate and females generally reporting better outcomes (25). However, the reasons are unclear, maybe because of the differences in genetic mutations, or hormonal and environmental factors (13, 22, 26, 27). Non-Hispanic astrocytoma and oligodendroglioma patients also showed poorer CSS. Regarding patient race, there was a significant difference in CSS. Specifically, Black astrocytoma patients showed greater CSS than White. There might be many variables that increase Black patients’ risk of survival, such as Black patients may be younger than White ones (7). Further analysis identified that Black patients were significantly younger than White patients, and interaction analysis of age and race excluded the statistical difference in survival, which supports the hypothesis. For different subtypes, astrocytoma has the worst prognosis, followed by mixed glioma, and oligodendroglioma has the best CSS. As expected, tumors with larger size (≥ 5 cm) and invasive behaviour tended to have poorer survival. Same to what Schiff et al. reviewed from literatures, increasing age, astrocytic histology, large tumor diameter (greater than 5 or 6 cm), tumors crossing midline, and the presence of neurologic deficits are the most important negative prognostic factors (22, 28).

Furthermore, a retrospective analysis of the prognosis of different treatment modalities showed that patients who underwent GTR had better CSS than STR, indicating that an important predictive factor in determining the survival of LGG patients may be the extent of surgical resection. Thereafter, surgery alone, surgery + CT, surgery + RT, or surgery + RT + CT reduced the death risk of LGG patients. However, patients who underwent surgery alone were associated with better survival than those who performed postoperative therapy, and CT even had a greater risk of mortality than no treatment. For CSS of three subtypes of LGGs, surgery alone reduced the death risk of all subtypes. In addition, surgery + RT decreased mortality risk of mixed glioma. However, astrocytoma patients who underwent CT and surgery + RT + CT have a higher mortality risk than no treatment. Similar to this, other investigations in LGGs based on CBTRUS or SEER have found either no change in survival with adjuvant therapy or a tendency toward poorer survival in patients receiving adjuvant therapy (2). This may be the unspecific information from the SEER database, which does not define the type of chemotherapy or radiation used or whether it was adjuvant or salvage treatment (29). Alternatively, since this is a retrospective study, doctors frequently choose high-risk LGGs patients for postoperative adjuvant radiotherapy and/or chemotherapy. Moreover, the side effects of postsurgical treatment, including fatigue, blood and bone marrow disorder, may also contribute to poorer survival (17). Currently, immediate postoperative radiotherapy and/or chemotherapy was thought benefit high-risk patients, whereas watchful waiting or observation is a reasonable option for low-risk patients (17). Therefore, it is critical for clinicians to distinguish high-risk and low-risk LGG patients.

In recent decades, numerous genetic anomalies that are strongly associated with prognosis and could serve as therapeutic targets for glioma have been discovered (30–33). Among these alterations, *IDH* mutations and 1p/19q codeletion are the two most important ones (33). Based on the limited information collected, we explored the impact of these alterations on LGG-specific survival. For astrocytoma patients with *IDH* mutations, oligodendroglioma with *IDH* mutations plus 1q/19q codeletion and LGGs with 1q/19q codeletion, have an 87.9%, 49.1% and 63.6% lower risk of death, respectively. Many studies also stated that the genetic alterations of LGGs play an important role in the prediction of tumor response to treatment, which is applied in the setting of chemotherapy or radiotherapy treatment regimens (34–37). To exclude the effect of treatment regimens in this study, the interaction effect of treatment regimens and molecular markers was considered. We observed that LGGs with 1q/19q co-deletion showed significantly longer survival by interaction analysis. Furthermore, after comparing the predictive efficiency of different patient demographics and tumor characteristics, the molecular alterations was one of the most important and robust prognostic factor with high ΔSHRs. These results indicated that molecular markers might play a principal role in identifying low-risk or high-risk LGG patients.

### 4.3 Limitations

The SEER database provides a large number of LGG patients and records cause-specific deaths, which helps us perform competing risk survival analyses to explore the risk factors. However, this study is limited by its retrospective nature, such as some key statistics cannot be measured, we cannot control exposure or outcome assessment, and instead must rely on others for accurate recordkeeping and so on. In detail, some information is unclear, such as it did not mention specific therapeutic drugs, the dose or mode of radiotherapy. In addition, the ICD-O-3 codes used in the present study and the WHO categories often used in the literature differ, which may affect the diagnostic bias of included LGG patients. Future research should be designed prospectively and incorporate more genetic risk, detailed surgery or adjuvant therapy information, and geographical demographics to improve our understanding of disparities in LGGs survival. It is also critical to stratify by many variables, such as age, sex, race, ethnicity, and geographical demographics, to identify variations in incidence and survival by these factors and socioeconomic status.

## 5 Conclusions

In the present study, the incidence trends of LGGs were updated. We observed that the IR of mixed gliomas decreased dramatically to nearly zero from 2013 to 2019, which demonstrated that with the development of molecular and cytogenetic criteria, the diagnosis accuracy is getting higher. Furthermore, the competing risk analysis was performed for LGG patients, and several risk factors were identified, including older age, male sex, the astrocytoma subtype, larger and more invasive tumor, and tumor residue after surgery, which correlated with worse survival. In addition, age (cut-off of 60 years), tumor invasiveness, and molecular markers are the most robust prognostic markers, which may help distinguish between high-risk and low-risk LGG patients. Based on the risk stratification, guideline-concordant treatments may help improve patient survival.

## Data availability statement

The original contributions presented in the study are included in the article/**Supplementary Material**. Further inquiries can be directed to the corresponding authors.

## Ethics statement

SEER database is publicly available and de-identified. Cases were collected from the SEER database and were analyzed anonymously in this study. Therefore, no ethical review or additional informed consent was required.

## Author contributions

Conceptualization, JC, HY, and XH; methodology, JC, WY, and ZZ; software, JC, WY, and ZZ; validation, JC, WY, and ZZ; formal analysis, JC and WY; investigation, JC, WY, and ZZ; resources, HY and XH; data curation, JC; writing-original draft preparation, JC; writing--review and editing, JC; visualization, JC; supervision, HY and XH; project administration, HY and XH; funding acquisition, HY and XH All authors have read and agreed to the published version of the manuscript. All authors contributed to the article and approved the submitted version.
