# Supplementary material for: Epidemiology and risk stratification of low-grade gliomas in the United States, 2004-2019: A competing-risk regression model for survival analysis
Source: Front Oncol. 2023 Mar 1;13:1079597. doi: 10.3389/fonc.2023.1079597 (PMC10014976; doi:10.3389/fonc.2023.1079597)
Supplement: Supplementary file 1 [file DataSheet_1.docx]

Supplementary Figures and Tables

# Supplementary Figure 1

**Supplementary Figure 1.** Overall survival analysis of low-grade glioma by Kaplan-Meier model (A) and multivariable Cox proportional hazards model (B).

#
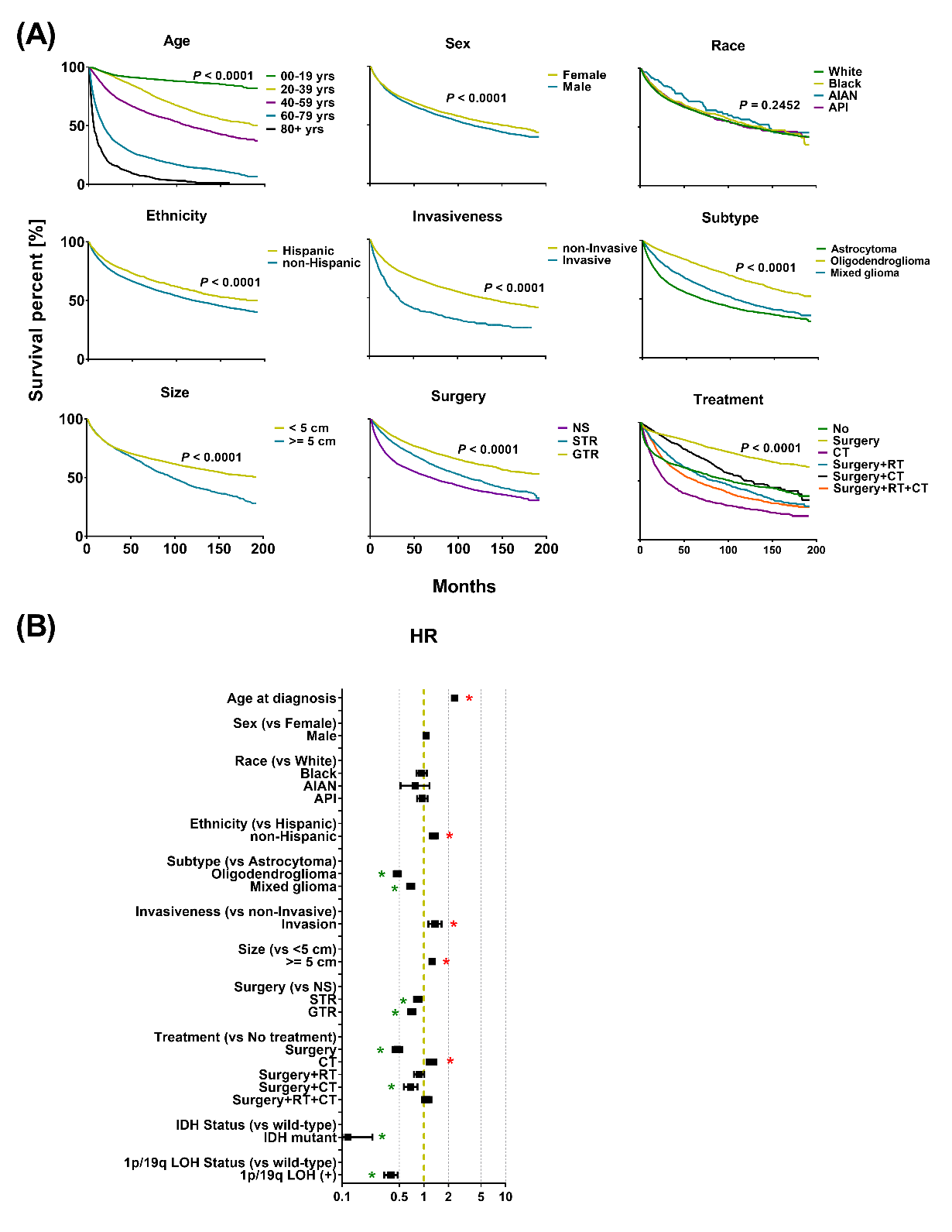


# 2. Supplementary Table 1. Baseline patient characteristics for astrocytoma, oligodendroglioma, and mixed glioma

|  |  | Astrocytoma | | Oligodendroglioma | | Mixed glioma | |
| --- | --- | --- | --- | --- | --- | --- | --- |
|  |  | Number % | | Number % | | Number % | |
| In total |  | 5245 | 39.33 | 3298 | 24.73 | 1890 | 14.17 |
| Age | 00-19 yrs | 722 | 13.77 | 176 | 5.34 | 87 | 4.60 |
|  | 20-39 yrs | 1477 | 28.16 | 1178 | 35.72 | 733 | 38.78 |
|  | 40-59 yrs | 1433 | 27.32 | 1431 | 43.39 | 746 | 39.47 |
|  | 60-79 yrs | 1327 | 25.30 | 466 | 14.13 | 295 | 15.61 |
|  | 80+ yrs | 286 | 5.45 | 47 | 1.43 | 29 | 1.53 |
| Sex | Female | 2291 | 43.68 | 1499 | 45.45 | 777 | 41.11 |
|  | Male | 2954 | 56.32 | 1799 | 54.55 | 1113 | 58.89 |
| Race | White | 4437 | 84.59 | 2864 | 86.84 | 1620 | 85.71 |
|  | Black | 395 | 7.53 | 150 | 4.55 | 91 | 4.81 |
|  | AIAN | 35 | 0.67 | 41 | 1.24 | 19 | 1.01 |
|  | API | 333 | 6.35 | 215 | 6.52 | 149 | 7.88 |
| Ethnicity | Hispanic | 809 | 15.37 | 551 | 16.71 | 324 | 17.14 |
|  | non-Hispanic | 4439 | 84.63 | 2474 | 75.02 | 1566 | 82.86 |
| Invasiveness | non-invasive | 3488 | 66.50 | 2455 | 74.44 | 1733 | 91.69 |
|  | Invasive | 183 | 3.49 | 21 | 0.64 | 63 | 3.33 |
| Size | < 5 cm | 2254 | 42.97 | 1398 | 42.39 | 758 | 40.11 |
|  | ≥ 5 cm | 1116 | 21.28 | 962 | 29.17 | 629 | 33.28 |
| Surgery | NS | 2882 | 54.95 | 1190 | 36.08 | 584 | 30.90 |
|  | STR | 1138 | 21.70 | 851 | 25.80 | 601 | 31.80 |
|  | GTR | 1157 | 22.06 | 1246 | 37.78 | 699 | 36.98 |
| Treatment | No | 1281 | 24.42 | 378 | 11.46 | 139 | 7.35 |
|  | Surgery | 1545 | 29.46 | 1422 | 43.12 | 566 | 29.95 |
|  | CT | 659 | 12.56 | 206 | 6.25 | 117 | 6.19 |
|  | Surgery + RT | 477 | 9.09 | 289 | 8.76 | 261 | 13.81 |
|  | Surgery + CT | 186 | 3.55 | 335 | 10.16 | 127 | 6.72 |
|  | Surgery + RT + CT | 1014 | 19.33 | 653 | 19.80 | 672 | 35.56 |

# Abbreviations: AIAN, American Indian/Alaska Native; API, Asian/Pacific Islander; CT, chemotherapy; GTR, gross total resection; No, no treatment; NS, no surgery; STR, subtotal resection; RT, radiotherapy.

# Supplementary Table 2. Sub-distribution hazard ratios (SHRs) and 95% confidence intervals (CIs) for LGG patients. Internal validation was performed by bootstrap analysis based on 1000 bootstrap samples.

| All LGGs | Subgroups | SHR | 95% CI | *P*-value | Bootstrap *P*-value |
| --- | --- | --- | --- | --- | --- |
| Age | 00-19 yrs | 1.985 (per 20yrs increase) | 1.967-2.003 | < 0.001 | 0.001 |
|  | 20-39 yrs |  |  |  |  |
|  | 40-59 yrs |  |  |  |  |
|  | 60-79 yrs |  |  |  |  |
|  | 80 + yrs |  |  |  |  |
| Sex | Female | Reference |  |  |  |
|  | Male | 1.111 | 1.096-1.126 | 0.0004 | 0.002 |
| Race | White | Reference |  |  |  |
|  | Black | 0.865 | 0.804-0.928 | 0.5369 | 0.942 |
|  | AIAN | 0.755 | 0.641-0.869 | 0.1271 | 0.721 |
|  | API | 0.997 | 0.938-1.056 | 0.0759 | 0.714 |
| Ethnicity | Hispanic | Reference |  |  |  |
|  | non-Hispanic | 1.342 | 1.320-1.364 | < 0.001 | 0.238 |
| Subtypes | Astrocytoma | Reference |  |  |  |
|  | Oligodendroglioma | 0.381 | 0.351-0.411 | < 0.001 | 0.001 |
|  | Mixed glioma | 0.76 | 0.732-0.788 | 0.099 | 0.713 |
| Invasiveness | non-Invasive | Reference |  |  |  |
|  | Invasive | 1.853 | 1.815-1.891 | < 0.001 | 0.001 |
| Size | < 5 cm | Reference |  |  |  |
|  | ≥ 5 cm | 1.262 | 1.244-1.280 | < 0.001 | 0.001 |
| Surgery Types | NS | Reference |  |  |  |
|  | STR | 0.703 | 0.6800.726 | 0.3528 | 0.020 |
|  | GTR | 0.464 | 0.440-0.488 | < 0.001 | 0.001 |
| Treatment Modality | No | Reference |  |  |  |
|  | Surgery | 0.377 | 0.346-0.408 | <0.001 | 0.001 |
|  | CT | 1.395 | 1.359-1.431 | <0.001 | 0.001 |
|  | Surgery + RT | 0.791 | 0.755-0.827 | 0.7527 | 0.758 |
|  | Surgery + CT | 0.578 | 0.528-0.628 | <0.001 | 0.685 |
|  | Surgery + RT+ CT | 0.954 | 0.927-0.981 | <0.001 | 0.003 |
| 1p/19q LOH status | 1p/19q LOH (-) | Reference |  |  |  |
|  | 1p/19q LOH (+) | 0.364 | 0.312-0.416 | <0.001 | 0.001 |

Continued Supplementary Table 1

| Astrocytoma | Subgroups | SHR | 95% CI | *P*-value | Bootstrap *P*-value |
| --- | --- | --- | --- | --- | --- |
| Age | 00-19 yrs | 1.921 (per 20yrs increase) | 1.899-1.943 | < 0.001 | 0.001 |
|  | 20-39 yrs |  |  |  |  |
|  | 40-59 yrs |  |  |  |  |
|  | 60-79 yrs |  |  |  |  |
|  | 80 + yrs |  |  |  |  |
| Sex | Female | Reference |  |  |  |
|  | Male | 1.0006 | 0.943-1.058 | 0.9187 | 0.879 |
| Race | White | Reference |  |  |  |
|  | Black | 0.658 | 0.536-0.780 | 0.0006 | 0.001 |
|  | AIAN | 0.925 | 0.589-1.261 | 0.8171 | 0.700 |
|  | API | 1.115 | 1.006-1.224 | 0.3185 | 0.370 |
| Ethnicity | Hispanic | Reference |  |  |  |
|  | non-Hispanic | 1.329 | 1.045-1.613 | 0.001 | 0.001 |
| Invasiveness | non-Invasive | Reference |  |  |  |
|  | Invasive | 1.298 | 1.157-1.439 | 0.0644 | 0.047 |
| Size | < 5 cm | Reference |  |  |  |
|  | ≥ 5 cm | 1.295 | 1.234-1.356 | <0.001 | 0.001 |
| Surgery Types | NS | Reference |  |  |  |
|  | STR | 0.767 | 0.685-0.849 | 0.0012 | 0.002 |
|  | GTR | 0.599 | 0.511-0.687 | <0.001 | 0.001 |
| Treatment Modality | No | Reference |  |  |  |
|  | Surgery | 0.592 | 0.487-0.697 | <0.001 | 0.001 |
|  | CT | 1.401 | 1.303-1.499 | 0.0006 | 0.002 |
|  | Surgery + RT | 1.152 | 1.048-1.256 | 0.1755 | 0.534 |
|  | Surgery + CT | 0.98 | 0.815-1.145 | 0.9009 | 0.570 |
|  | Surgery + RT+ CT | 1.54 | 1.445-1.635 | <0.001 | 0.002 |
| IDH status | IDH wild-type | Reference |  |  |  |
|  | IDH mutant | 0.121 | 0.001-0.305 | <0.001 | 0.001 |

Continued Supplementary Table 1

| Oligodendrogliomas | Subgroups | SHR | 95% CI | *P*-value | Bootstrap *P*-value |
| --- | --- | --- | --- | --- | --- |
| Age | 00-19 yrs | 1.869 (per 20yrs increase) | 1.821-1.917 | < 0.001 | 0.001 |
|  | 20-39 yrs |  |  |  |  |
|  | 40-59 yrs |  |  |  |  |
|  | 60-79 yrs |  |  |  |  |
|  | 80 + yrs |  |  |  |  |
| Sex | Female | Reference |  |  |  |
|  | Male | 1.235 | 1.141-1.329 | 0.0242 | 0.041 |
| Race | White | Reference |  |  |  |
|  | Black | 1.206 | 1.002-1.410 | 0.3588 | 0.251 |
|  | AIAN | 0.718 | 0.323-1.113 | 0.4003 | 0.784 |
|  | API | 0.83 | 0.634-1.026 | 0.3409 | 0.213 |
| Ethnicity | Hispanic | Reference |  |  |  |
|  | non-Hispanic | 1.522 | 1.374-1.670 | 0.0046 | 0.391 |
| Invasiveness | non-Invasive | Reference |  |  |  |
|  | Invasive | 2.108 | 1.883-2.333 | 0.0009 | 0.005 |
| Size | < 5 cm | Reference |  |  |  |
|  | ≥ 5 cm | 1.468 | 1.370-1.566 | <0.001 | 0.001 |
| Surgery Types | NS | Reference |  |  |  |
|  | STR | 0.803 | 0.672-0.934 | 0.0932 | 0.022 |
|  | GTR | 0.664 | 0.537-0.791 | 0.0013 | 0.001 |
| Treatment Modality | No | Reference |  |  |  |
|  | Surgery | 0.592 | 0.417-0.768 | 0.0028 | 0.007 |
|  | CT | 1.029 | 0.812-1.246 | 0.8938 | 0.001 |
|  | Surgery + RT | 1.411 | 1.224-1.598 | 0.0656 | 0.352 |
|  | Surgery + CT | 0.878 | 0.683-1.073 | 0.5045 | 0.002 |
|  | Surgery + RT+ CT | 1.489 | 1.294-1.684 | 0.0416 | 0.001 |
| IDH and LOH status | Unmatched | Reference |  |  |  |
|  | IDH mutant +  1p/19q LOH (+) | 0.509 | 0.339-0.679 | 0.0474 | 0.066 |

Continued Supplementary Table 1

| Mixed gliomas | Subgroups | SHR | 95% CI | *P*-value | Bootstrap *P*-value |
| --- | --- | --- | --- | --- | --- |
| Age | 00-19 yrs | 1.784 (per 20yrs increase) | 1.205-2.363 | < 0.001 | 0.001 |
|  | 20-39 yrs |  |  |  |  |
|  | 40-59 yrs |  |  |  |  |
|  | 60-79 yrs |  |  |  |  |
|  | 80 + yrs |  |  |  |  |
| Sex | Female | Reference |  |  |  |
|  | Male | 0.956 | 0.871-1.041 | 0.6004 | 0.366 |
| Race | White | Reference |  |  |  |
|  | Black | 1.234 | 1.056-1.412 | 0.238 | 0.061 |
|  | AIAN | 0.723 | 0.265-1.181 | 0.478 | 0.897 |
|  | API | 0.793 | 0.622-0.964 | 0.1752 | 0.216 |
| Ethnicity | Hispanic | Reference |  |  |  |
|  | non-Hispanic | 1.145 | 1.028-1.262 | 0.2482 | 0.574 |
| Invasiveness | non-Invasive | Reference |  |  |  |
|  | Invasive | 2.051 | 1.846-2.256 | 0.0005 | 0.029 |
| Size | < 5 cm | Reference |  |  |  |
|  | ≥ 5 cm | 1.204 | 1.118-1.290 | 0.0312 | 0.008 |
| Surgery Types | NS | Reference |  |  |  |
|  | STR | 0.887 | 0.758-1.016 | 0.3543 | 0.804 |
|  | GTR | 0.824 | 0.695-0.953 | 0.1331 | 0.929 |
| Treatment Modality | No | Reference |  |  |  |
|  | Surgery | 0.409 | 0.206-0.612 | <0.001 | 0.007 |
|  | CT | 1.071 | 0.845-1.297 | 0.762 | 0.276 |
|  | Surgery + RT | 0.573 | 0.361-0.785 | 0.0086 | 0.039 |
|  | Surgery + CT | 0.583 | 0.348-0.818 | 0.0216 | 0.139 |
|  | Surgery + RT+ CT | 0.821 | 0.625-1.017 | 0.3156 | 0.448 |

Abbreviations: AIAN, American Indian/Alaska Native; API, Asian/Pacific Islander; CT, chemotherapy; GTR, gross total resection; No, no treatment; NS, no surgery; STR, subtotal resection; RT, radiotherapy.
